# Retroperitoneal Cystic Nodal Metastasis of Renal Cell Carcinoma

**DOI:** 10.1155/2018/1605102

**Published:** 2018-05-03

**Authors:** Takahisa Yamashita, Makoto Morozumi, Morihiro Higashi, Shuji Momose, Jun-ichi Tamaru

**Affiliations:** ^1^Department of Urology, Saitama Medical Center, Saitama Medical University, 1981 Kamoda, Kawagoe-shi, Saitama 3508550, Japan; ^2^Department of Pathology, Saitama Medical Center, Saitama Medical University, 1981 Kamoda, Kawagoe-shi, Saitama 3508550, Japan

## Abstract

Cystic nodal metastasis of renal cell carcinoma is very rare. The pathogenesis of cystic nodal metastasis is thought to involve obstruction of a lymphoid vessel draining the kidney by tumor cells and retrograde metastasis from the primary site to the lymph node along the lymphatic vessels. In this study, a surgical case of small renal cell carcinoma with retroperitoneal cystic nodal metastasis is reported.

## 1. Introduction

Approximately 18% of patients with renal cell carcinoma (RCC) have metastasis at diagnosis [1], and lymph node metastasis appears in 22% of all patients with metastatic RCC [2]. Cases of RCC with lymph node metastasis have low response rates to immunotherapy and a poor prognosis compared to those without lymph node metastasis. A case of small renal cell carcinoma with retroperitoneal cystic nodal metastasis is presented. Cystic nodal metastasis is often seen in head and neck cancers, but it is very rare in renal cell carcinoma. In this report, a surgical case of retroperitoneal cystic nodal metastasis of RCC is described. The patient provided his written informed consent for this study.

## 2. Case Presentation

**Figure 1:**
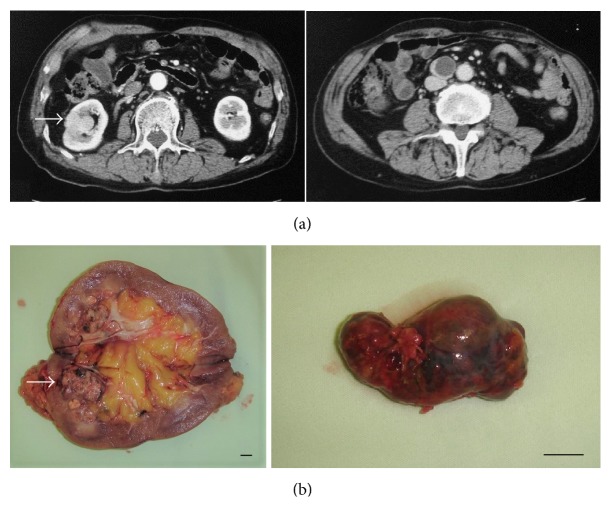
(a) Enhanced CT of the abdomen showing a nonexophytic and hypervascular tumor (arrow) in the right kidney (left) and a cystic lymph node in the aortocaval area (right). (b) Macroscopic findings of the resected specimens showing an ash-colored, tessellated, and fibrous capsuled renal tumor (arrow) in the right kidney (left) and a tense and cystic lymph node (right); bars, 1 cm.

**Figure 2:**
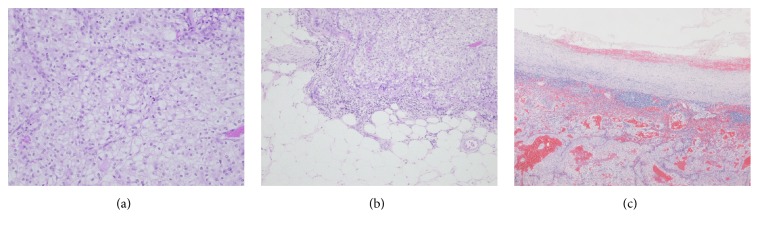
The cancer nests of the epithelial cells with a clear cytoplasm and a distinct cell membrane, separated by many capillary vessels (a) and perinephric fat invasion in the renal tumor (b). The same histological nests are seen in the renal tumor and the cystic lymph node (c).

**Figure 3:**
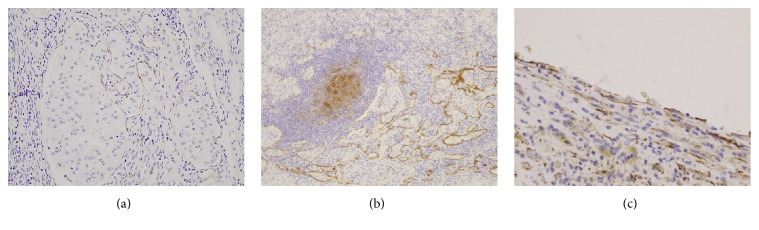
Immunohistochemical findings showing many lymphatic ducts expressing D2-40 filled with cancer nests in the kidney (a) and lymph node (b) and the coated cell of the cystic wall expressing D2-40 (c).

A 74-year-old Japanese man underwent a physical examination at another hospital, and a retroperitoneal cystic mass (37 mm in diameter), which was located at the aortocaval region, was seen on transabdominal ultrasonographic examination. He had taken oral immunosuppressive agents for a long time for rheumatoid arthritis, and his score of Karnofsky performance status was 70. In our institution, CT showed not only an enlarged and cystic aortocaval node (25 mm × 25 mm), which was located lower than the right kidney, but also a nonexophytic and hypervascular renal tumor (30 mm × 27 mm) (Figure 1). There was no space occupied lesion in the bilateral lung. Notable laboratory values were WBC 10,600/*μ*l, CRP 3.0 mg/dl, and SIL-2R (soluble IL-2 receptor) 1350 ng/ml. Since it could not be determined whether the cystic lymph node was caused by cancer metastasis, malignant lymphoma, or inflammatory disease, the patient underwent right radical nephrectomy with retroperitoneal localized lymphoidectomy in consideration for his general status. Macroscopic examination of the resected specimens showed an ash-colored, tessellated, and fibrous capsuled renal tumor and a tense and cystic lymph node (Figure 1). Histopathological examination showed nests of atypical epithelial cells with clear cytoplasm and a distinct cell membrane, separated by many capillary vessels in the renal tumor. Hence, the renal tumor was diagnosed as a clear cell RCC (Grade 2). Moreover, there were both microvessel and lymphatic invasion, as well as renal sinus and perinephric fat invasion in the kidney. On the other hand, in the cystic lymph node, there were the same features as the right renal tumor and a fibrous cystic wall (Figure 2). The final diagnosis was locally invasive RCC with retroperitoneal lymph node metastasis (pT3a, pN2 (2/2), cM0). Furthermore, immunohistochemical examination showed that the many lymphatic ducts expressing D2-40 were filled with cancer nests in both the kidney and the lymph node, and a few coated cells of the cystic nodal wall expressed D2-40 (Figure 3). He was not treated with adjuvant therapy because of his general status and mind, and fortunately, there was no evidence of the recurrence and metastasis at 6 months after the surgery.

## 3. Discussion

Lymph node metastasis is the third most common site of metastatic RCC, occurring in 22% of cases. However, cystic nodal metastasis of RCC is very rare, and only three cases have been previously reported in the literature [3–5]. It is known that cystic nodal metastasis of RCC is caused by obstruction of the lymphatic vessels draining the kidney by tumor cells and retrograde metastasis from the primary site to the lymph nodes along the lymphatic vessels. In the current case, many areas of lymphatic invasion were seen in the primary tumor, and the cystic wall in the lymph node was covered with lymphatic vessels that expressed D2-40. Moreover, the cystic lymph node was located lower than the right kidney. Therefore, these pathological findings of the current case are compatible with the pathogenesis of cystic nodal metastasis previously discussed. Moreover, lymphatic invasion and cystic nodal metastasis were confirmed, although the primary tumor size was <4 cm. Taken together, regardless of the tumor size, there is a possibility that a retroperitoneal cystic mass with a renal tumor can be a lymph node metastasis of RCC caused by obstruction of the lymphatic vessels draining the kidney by tumor cells, and if surgical treatment is considered, retroperitoneal lymphoidectomy as well as nephrectomy may be performed.

## 4. Conclusion

A retroperitoneal cystic nodal metastasis must be considered in a patient having a renal tumor with a retroperitoneal cystic mass, regardless of the tumor size.
